# Post-surgical Median Arcuate Ligament Syndrome (MALS) Symptom Exacerbation Treated With Osteopathic Manipulative Treatment (OMT): A Case Report

**DOI:** 10.7759/cureus.61509

**Published:** 2024-06-01

**Authors:** Mikhail Volokitin, Amanda Bergman

**Affiliations:** 1 Osteopathic Manipulative Medicine, Touro College of Osteopathic Medicine, New York, USA

**Keywords:** celiac artery compression syndrome, median arcuate ligament syndrome, harjola-marable syndrome, celiac trunk compression syndrome, osteopathic manipulative treatment (omt), osteopathic manipulative medicine, dunbar syndrome, celiac axis syndrome

## Abstract

Median arcuate ligament syndrome (MALS, also known as celiac artery compression syndrome, celiac axis syndrome, celiac trunk compression syndrome, Dunbar syndrome, or Harjola-Marable syndrome) is a rare condition characterized by abdominal pain attributed to the compression of the celiac artery and celiac ganglia by the median arcuate ligament. Pain can occur post-prandially and may be accompanied by weight loss, nausea, or vomiting. Following angiographic diagnosis, current definitive treatment may include open or laparoscopic decompression surgery with celiac ganglion removal (if affected), which has been found to provide relief. In this case report, we outline a young female patient with a MALS diagnosis and subsequent surgery, but whose pain recurred in various stress-related instances even after surgical intervention. After a particular pain episode, osteopathic manipulative treatment (OMT) was applied, with a focus on restoring autonomic balance through the use of various gentle osteopathic treatment techniques. A significant reduction in pain was reported post-treatment, followed by complete pain resolution, indicating a great benefit to the incorporation of OMT into the treatment plan of MALS patients in future osteopathic practice.

## Introduction

The thoracoabdominal diaphragm is anchored in great part due to the median arcuate ligament (MAL), formed by the union of the left and right diaphragmatic crura at the anterosuperior border of the aortic hiatus [1], with an attachment posteriorly to the body of L1 or L2 [2]. While the celiac trunk is normally inferior to the MAL, the celiac artery or MAL may be anatomically mispositioned and can lead to the compression of the celiac artery by the MAL. Such a phenomenon is known as median arcuate ligament syndrome (MALS) [3], a rare finding with an incidence rate of two in 100,000 [4]. Compression of the celiac plexus by the MAL may also occur in MALS due to their close proximity, leading to a further exacerbation of symptoms [1].

MALS is often a diagnosis of exclusion, with symptoms that may include generalized or post-prandial abdominal pain, nausea, vomiting, and/or weight loss [5,6]. Diagnosis is made via computed tomography angiogram (CTA) or magnetic resonance angiography (MRA), with the definitive treatment of open or laparoscopic decompression surgery which may include celiac ganglion removal or ablation to address neuropathic pain [7]. 

As an adjunct or alternative treatment to surgery, osteopathic manipulative treatment (OMT) techniques may be used in MALS to effectively decrease pain and neurovascular congestion, as described in a previous study [1]. However, possible lingering effects of MALS post-operatively are scarcely documented. As such, the following case report describes the successful osteopathic treatment of a symptom exacerbation in a post-surgical MALS patient, shedding light on the effectiveness of OMT in the diagnosis and treatment of visceral-associated pain through the restoration of autonomic balance.

## Case presentation

This case report follows a 23-year-old Caucasian female with a past medical history of hypothyroidism controlled with levothyroxine and liothyronine and constipation managed with linaclotide.

Leading up to her MALS diagnosis, the patient suffered from severe upper epigastric pain since 2013, described as an intense pressure present at baseline, exacerbated post-prandially and often accompanied by nausea. After a series of negative tests, including endoscopies, upper gastrointestinal series, and nuclear imaging, the patient attempted multiple dietary changes which failed to improve symptoms.

**Figure 1:**
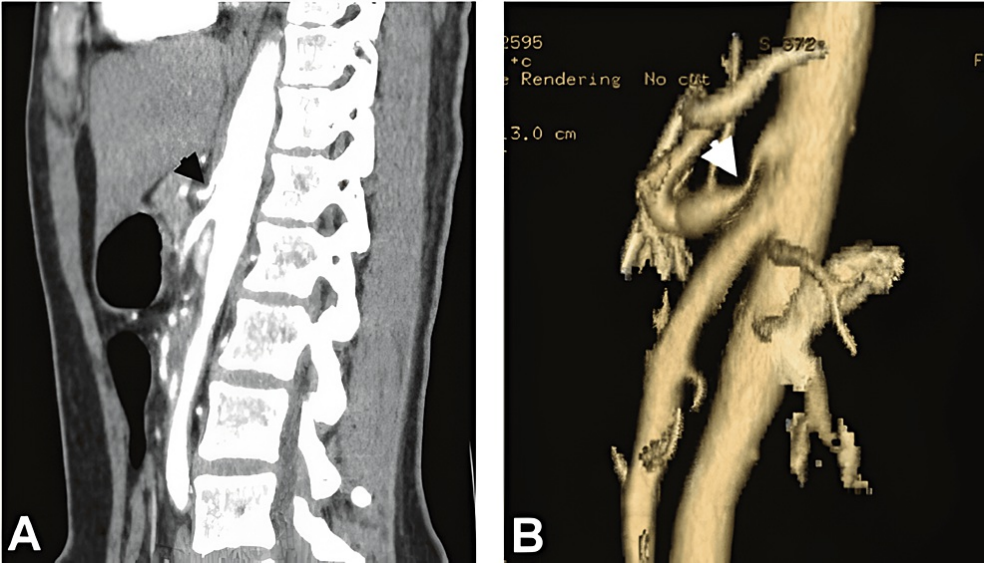
2D and 3D representations of a CTA revealing stenosis of the proximal celiac artery (A black arrow, B white arrow), establishing a diagnosis of MALS. CTA: computed tomography angiogram; MALS: median arcuate ligament syndrome Figure Source: Chou et al., 2012 [8]; reproduced under the Creative Commons Attribution License (CC BY 3.0 Deed)

In 2018, a MALS diagnosis was confirmed via vascular Doppler ultrasonography, which revealed >70% stenosis in the celiac artery and proximal celiac trunk. Furthermore, subsequent MRA revealed compression of the celiac artery by the MAL. Two weeks post-diagnosis, the patient underwent a celiac plexus nerve block and began a pain management regimen of gabapentin and duloxetine. Due to continued pain in late 2018, the patient underwent an elective laparoscopic MAL decompression surgery, with the removal of surrounding inflamed nerves (Figure 1).

While noting improvement in her pain beginning three months post-operation, the patient since reported "flare-up" episodes of upper epigastric pain, resembling her initial MALS symptoms. Particularly, after the loss of a family member in May 2023 (a time of reported high stress), the patient experienced a week-long "flare-up" episode with a severe exacerbation of symptoms, leading her to seek osteopathic treatment for pain relief. 

An osteopathic structural exam revealed a positive erythema test at T5-T9 on the left in a fusiform distribution with warmth on palpation, indicating viscerosomatic dysfunction. Left erector spinae hypertonicity and fullness were appreciated at T5-T9, as well as suboccipital muscle fullness, tenderness, and hypertonicity. Additionally, a Chapman's point was found at the left sixth intercostal space, with confirmatory tenderness at the left T6 transverse process posteriorly. Celiac ganglion (T5-T9) fascial restriction was found with associated severe epigastric tenderness on palpation, indicating viscerosomatic dysfunction.

After obtaining consent, treatment techniques performed included bilateral rib raising to the T5-T9 region, myofascial release to the hypertonic suboccipital and midthoracic musculature, treatment of the localized Chapman's point via application of pressure in a circular motion, and treatment of the restricted celiac plexus fascia via collateral ganglia release with respiratory assist.

Immediately following treatment, somatic dysfunctions were reassessed, and a softening of the manipulated tissues was noted. The patient was advised to stay hydrated and take NSAIDs if necessary, if pain worsened after treatment. Two days after treatment, the patient reported significant pain reduction which fully resolved shortly thereafter. A follow-up exam revealed normalization of previously positive findings, and the patient reported post-treatment pain relief that lasted several months.

## Discussion

**Figure 2:**
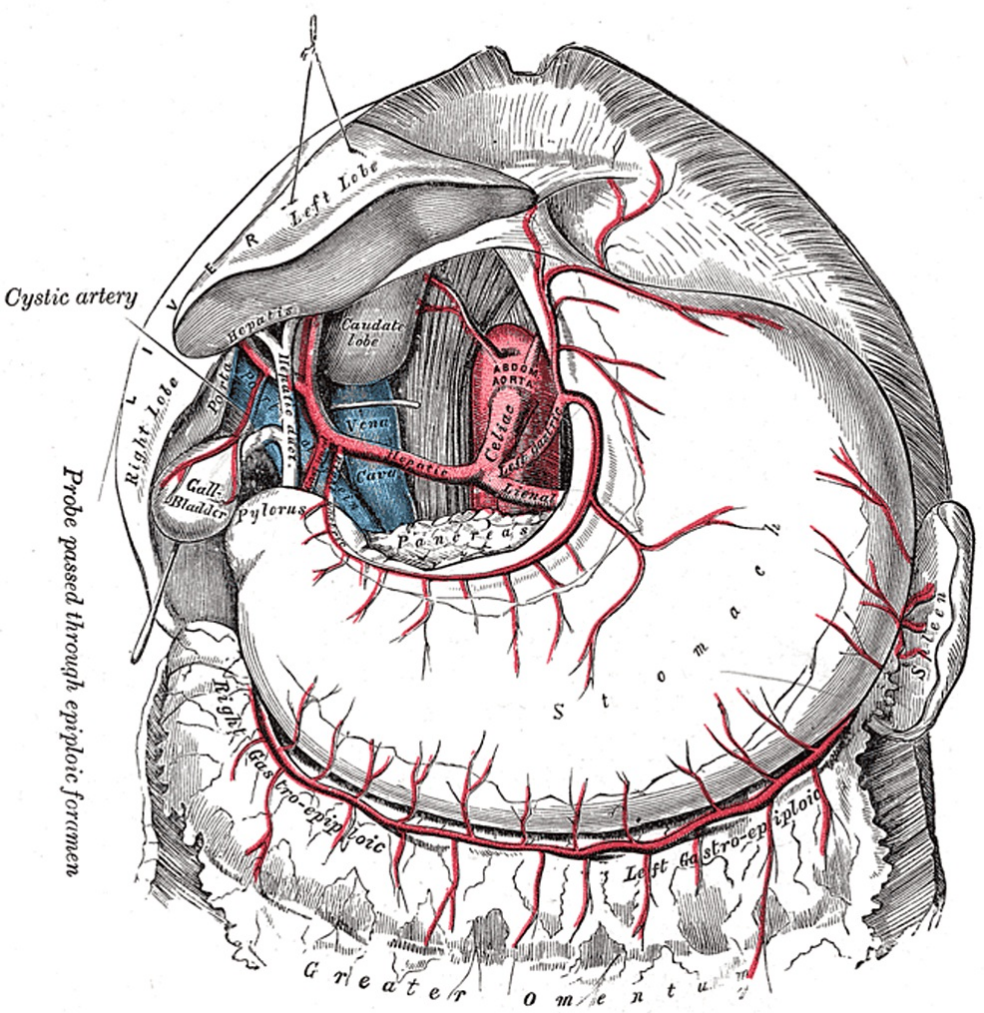
The celiac artery and its blood supply to the stomach, spleen, and liver. Figure Source: [9]; In the public domain by CC BY-SA 4.0

The compression of the celiac trunk in MALS may lead to compromise of the organs it supplies, including the stomach, spleen, and liver (Figure 2).

As less blood flow is supplied due to the compression, symptoms are often felt post-prandially (when more blood supply is needed) [1], as was noted in this patient. While 85% of patients reported immediate relief from symptoms post-operatively [10], this case is unique in that it describes a patient with repeated episodes of symptoms even after a successful surgical treatment. A possible explanation for this occurrence may be that the ischemic changes caused by the celiac artery compression and nerve irritation in this patient led to sympathetic nerve fiber hypersensitivity and a lasting lower pain threshold [11] susceptible to various triggers. Stress in particular, as experienced by this patient, may be a possible trigger of this autonomic imbalance and subsequent pain response, as supported by a previous study on stress-induced visceral pain in which stress was shown to increase the gene expression of pro-nociceptive neurotransmitters [12]. 

**Figure 3:**
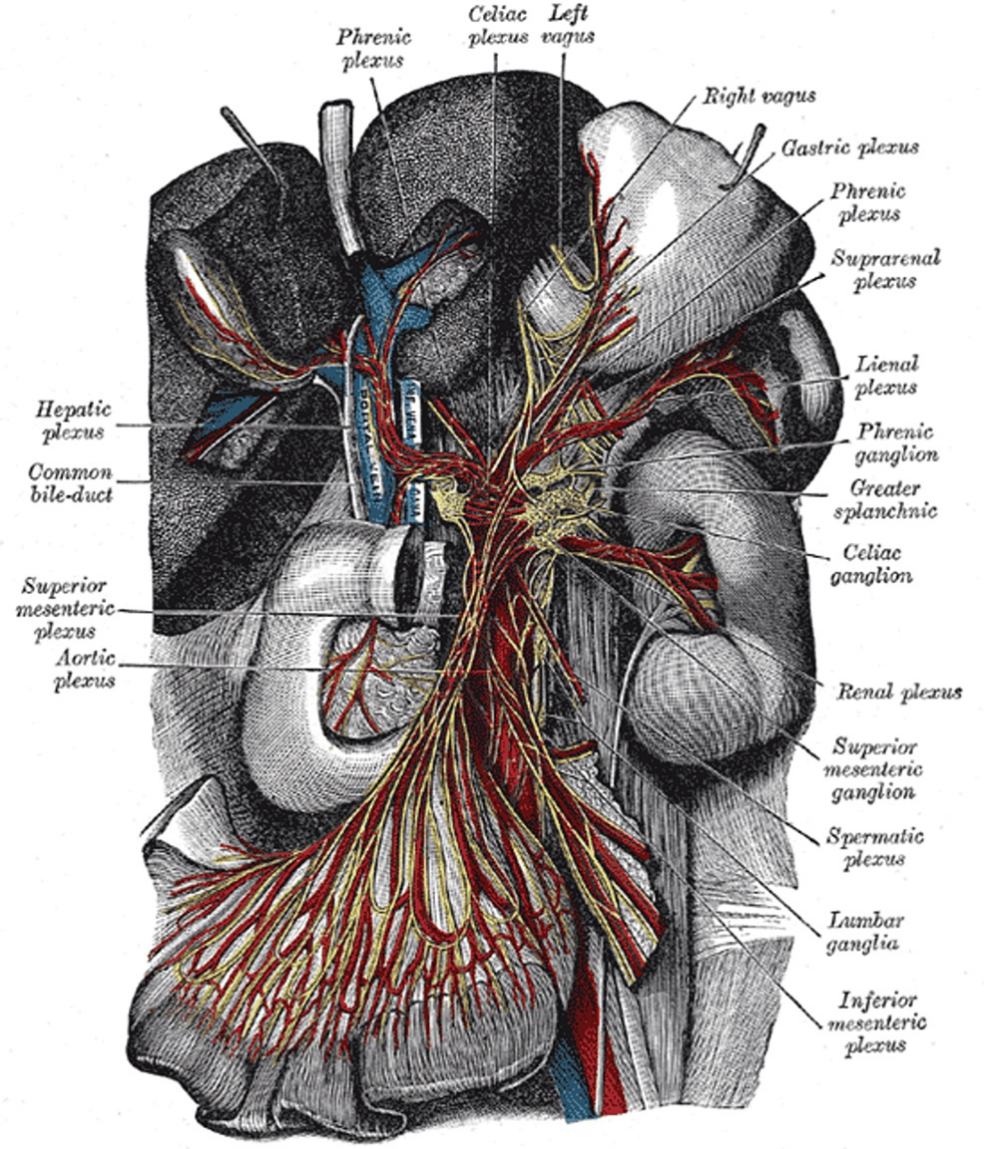
The celiac plexus and surrounding vasculature. Figure Source: [13]; In the public domain by CC BY-SA 4.0

Critical to the proper functioning of the digestive system is a well-balanced autonomic nervous system, which can either help or hinder its intrinsic function. The stomach, spleen, and liver receive parasympathetic innervation from the vagus nerve, increasing gastrointestinal motility when activated. On the other hand, the celiac plexus is the main contributor to the sympathetic innervation of the gastrointestinal organs and decreases gastrointestinal motility when activated (Figure 3) [11].

As seen in this MALS patient, an imbalance in the sympathetic and parasympathetic nervous systems may result from an overactive sympathetic response caused by the compression of the celiac plexus. This anatomical compression leads to a decrease in myoelectric activity and nerve functionality of the stomach and hinders its contractile rhythm, as demonstrated in a previous study [14].

As expected, palpable changes to the celiac plexus were noted on the osteopathic structural exam of this patient, with hypertonicity and tenderness found over the linea alba inferior to the xiphoid process, indicating sympathetic overstimulation. Sympathetic dysfunction was further reflected by the presence of a Chapman's point, a neurolymphatic reflex formed by "excessive sympathetic tone from an irritated, diseased, or stressed organ" leading to lymphatic stasis which manifests as a tender myofascial nodule at a specified location corresponding to that organ [15]. A Chapman's point was localized to the patient's left sixth intercostal space anteriorly and the transverse process of T6 posteriorly, corresponding to a component of the sympathetic innervation to the stomach. Additionally, inflammation or irritation of an organ (such as the stomach in the case of MALS) can stimulate visceral afferent sympathetic nerve fibers, which may initiate a long reflex with nociceptive information traveling through the dorsal spinal root and horn to interneurons which ultimately innervate both visceral and somatic structures located at the same spinal level. Such a viscerosomatic reflex was noted in this patient at spinal level T5-T9, corresponding to the segmental levels innervating the stomach, and was recognized by a positive erythema test at this location, with increased redness and irritation to the T5-T9 spinal levels on the left side, in addition to palpable tissue hypertonicity, warmth, and decreased mobility [16].

Taking these physical findings into account, multiple osteopathic techniques were selected and performed in an effort to restore gastrointestinal autonomic balance in this patient, including rib raising performed to the region of T5-T9 to reduce sympathetic tone, treatment of the hypertonic suboccipital musculature to promote a parasympathetic response via improved functioning of the vagus nerve located deep to these structures [17], treatment of the taut celiac plexus and the localized Chapman's point, as well as treatment of the T5-T9 somatic dysfunctions and muscle hypertonicity posteriorly. Given the patient's improvement of symptoms in the days following treatment, it appears that the patient responded positively to the autonomic-targeted techniques performed.

## Conclusions

We present a case of a patient with MALS who continued to experience original symptoms after an attempt at surgical correction. OMT directed to restore autonomic balance, improve gastrointestinal functionality, and normalize affected muscle tone was shown to be successful, as demonstrated by the patient's symptomatic improvement post-treatment. Utilization of the applied techniques along with osteopathic philosophy including taking a whole-person approach may be used in similar cases as an additional approach to management.
